# Application of tri-colour, dual fusion fluorescence in situ hybridization (FISH) system for the characterization of BCR-ABL1 fusion in chronic myelogenous leukaemia (CML) and residual disease monitoring

**DOI:** 10.1186/1471-2326-9-4

**Published:** 2009-07-07

**Authors:** Lisa LP Siu, Edmond SK Ma, Wai Shan Wong, Man Hong Chan, Kit Fai Wong

**Affiliations:** 1 Department of Pathology, Queen Elizabeth Hospital, Hong Kong SAR, PR China; 2 Division of Molecular Pathology, Department of Pathology, Hong Kong Sanatorium & Hospital, Hong Kong SAR, PR China; 3 Department of Medicine, Queen Elizabeth Hospital, Hong Kong SAR, PR China

## Abstract

**Background:**

We studied the application of the *BCR-ABL1 *+ 9q34 tri-colour dual fusion fluorescence *in situ *hybridization (FISH) system in the characterization of fusion signal pattern and the monitoring of residual disease in chronic myelogenous leukaemia (CML). The signal constellation on metaphases with the tri-colour dual fusion system was defined. The knowledge of various signal patterns obtained from the different genetic rearrangements was further applied to the analysis of hybridization signals on interphase nuclei.

**Methods:**

*BCR-ABL1 *dual colour, dual fusion FISH (D-FISH) was performed on diagnostic samples of 22 CML patients. The tri-colour FISH system was performed on cases that showed aberrant signal patterns other than the classical 1 green (G) 1 orange (O) 2 fusions (F). Using the aqua band-pass filter, random signal overlap in interphase nuclei would be indicated by the presence of an aqua signal (*ASS1*), while genuine fusion was represented by the absence of the *ASS1 *signal.

**Results:**

Using the D-FISH system, the signal patterns could be categorized into 4 groups: group 1 (n = 17) showed the classical 1G1O2F; group 2 (n = 2) showed 2G1O1F indicating *ABL1 *deletion; group 3 (n = 1) showed 1G2O1F indicating *BCR *deletion; group 4 (n = 2) with 1G1O1F indicating reciprocal *ABL1-BCR *deletion. The tri-colour dual fusion system correlated with the D-FISH system for cases with der(9) deletion. The added aqua-labelled *ASS1 *probe was useful in differentiating random signal overlap from genuine *BCR-ABL1 *fusion in the interphase cells (group 4).

**Conclusion:**

Although the D-FISH probe was valuable in establishing the different patterns of aberrant signals and monitoring patients with the classic 2-fusion signals in CML, the tri-colour dual fusion probe should be used for patients with der(9) deletion to monitor response to treatment.

## Background

Chronic myelogenous leukaemia (CML) is a clonal haematopoietic stem cell disorder characterized by the translocation t(9;22)(q34;q11.2). The translocation fuses the 5' sequences of the *BCR *gene on chromosome 22 with the 3' sequences of the *ABL1 *gene on the chromosome 9. The dual colour, dual fusion fluorescence *in situ *hybridization (D-FISH) system can demonstrate the *BCR-ABL1 *fusion [1]. In classical t(9;22), there is one signal each for the wild type alleles and two fusion signals, one for the fusion gene and the other for the reciprocal product in a positive cell. This dual fusion pattern has provided good analytical sensitivity for the monitoring of disease response to therapy. However, around 15% of CML patients show deletion of the reciprocal *ABL1-BCR *fusion on the derivative chromosome 9 (der(9) deletion), often with loss of both chromosomes 9 and 22 sequences on either side of the breakpoint [2-4]. The der(9) deletion is associated with an inferior outcome when compared with those without such deletion, but the poor prognosis may at least be partially abrogated by imatinib therapy [5]. In the presence of der(9) deletion, false positivity is a problem with the use of dual colour single or even dual fusion probes. In this study, we have used metaphase FISH to study the signal pattern on diagnostic samples of 22 CML patients. In addition, the *BCR-ABL1 *+ 9q34 tri-colour, dual fusion system was applied on all cases with atypical D-FISH pattern and also in the monitoring of residual disease in a patient with der(9) deletion.

## Methods

The diagnostic samples, including both peripheral blood and bone marrow from 22 CML patients were retrieved for the study. Cytogenetics study on all the samples showed t(9;22)(q34;q11.2). *BCR-ABL1 *dual colour dual fusion FISH (D-FISH) (Vysis, Abbott Molecular Inc., IL) was performed on cell pellets kept in Carnoy's fixative that were previously processed for cytogenetics analysis. For cases with aberrant signal pattern other than the classical 1 green (G), 1 orange (O) and 2 fusions (F), the *BCR-ABL1 *+ 9q34 tri-colour dual fusion FISH (Vysis) system was applied to further characterize the signal pattern.

Briefly, cells were dropped onto microscopic slides and aged overnight at 65°C before performing the FISH hybridization procedures according to manufacturer's protocol. 10 μl of the probe mixture was applied to a 18 × 18 mm hybridization area. The hybridized area was sealed with rubber cement to avoid drying in subsequent denaturation and hybridization procedure. The chromosomes and probes were co-denatured at 75°C for 5 minutes and allowed to hybridize overnight at 37°C (Hybridizer, DakoCytomation). Slides were washed in 0.4× SSC/0.3% NP40 at 70°C for 2 minutes followed by 2× SSC/0.1% NP40 at room temperature for 1 minute. The wash step was necessary to remove non-specific hybridization signals. Slides were then counterstained and mounted with 10 μl 4',6-diamidino-2-phenylindole (DAPI II, 125 ng/ml, Vysis).

For each hybridization experiment, 200 interphase cells were examined under oil immersion at 1000× magnification using the Nikon (model 80i) fluorescence microscope. The FISH signals were analyzed with fluorochrome-specific single band-pass filters for the *BCR *(SpectrumGreen signals) and *ABL1 *(SpectrumOrange signals). The *BCR-ABL1 *fusion signals were scored with a dual band-pass filter. For the tri-colour dual fusion system, cells were further examined using a single band-pass filter specific for the *ASS1 *(SpectrumAqua signals). Signal patterns in both interphase and metaphase were characterized using both D-FISH and tri-colour dual fusion FISH system. The Cytovision imaging workstation (Version 2.7) was used for image capture and processing.

This study was performed according to the regulation set by the Ethics Committee of Queen Elizabeth Hospital.

## Results

**Figure 1:**
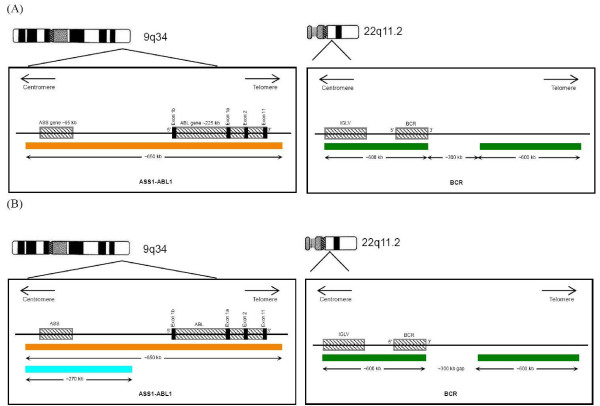
**FISH Probe configuration**. (A) BCR-ABL1 dual colour, dual fusion (D-FISH). (B) BCR-ABL1 + 9q34 tri-colour, dual fusion.

The probe configuration for both the D-FISH and the tri-colour dual fusion FISH were shown in Figure 1. In classical t(9;22), there was fusion of the *BCR *on chromosome 22 with the *ABL1 *on chromosome 9. The D-FISH system showed individual orange and green signals from the normal 9 and 22 chromosomes and two orange/green fusion (F) signals, one each from the derivative 9 and 22 chromosomes. In the tri-colour dual fusion system, an additional *ASS1 *gene (centromeric to the *ABL1*) on chromosome 9 was labeled with SpectrumAqua. The probe target on the normal chromosomes 9 and 22 was expected to produce single orange/aqua and green signals respectively. Thus, the der(22) was visible as an orange/green fusion signal without an associated aqua signal. The der(9) had an orange/aqua/green fusion signal. Since the *ASS1 *was not involved in the translocation, the aqua signal was present when there was random juxtaposition of chromosome 9 and 22 but not in genuine *BCR-ABL1 *fusion.

**Figure 2:**
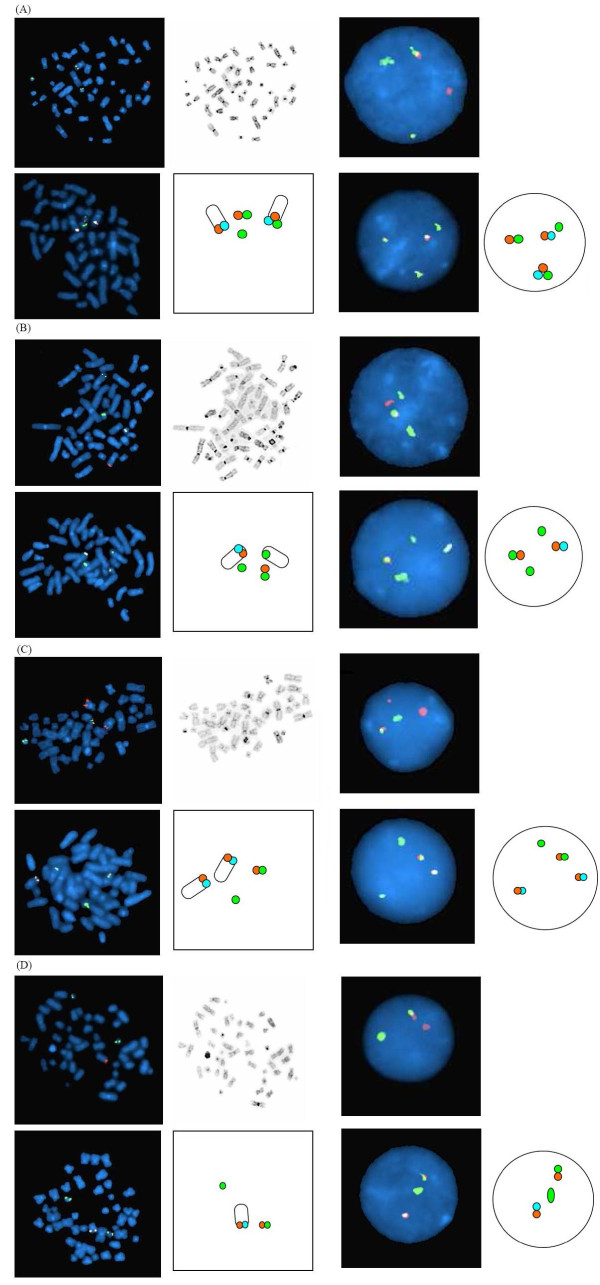
**Representative interphase and metaphase images from the four pattern of signals**. The upper panels are probe and reverse DAPI images using the BCR-ABL1 dual colour, dual fusion (D-FISH). The lower panels are probe and the cartoon images using the BCR-ABL1 tri-colour, dual fusion. (A) Group 1: classical 1G1O2F signal pattern using D-FISH. (B) Group 2:*ABL1 *deletion, 2G1O1F signal pattern using D-FISH. (C) Group 3: *BCR *deletion, 1G2O1F signal pattern using D-FISH. (D) Group 4: reciprocal *ABL1-BCR *deletion, 1G1O1F signal pattern using D-FISH.

Using the D-FISH system, our patients could be categorized into 4 groups (Figure 2, A – D, upper panel): group 1 (n = 17) showed the classical pattern of 1G1O2F; group 2 (n = 2) showed 2G1O1F indicating *ABL1 *deletion; group 3 (n = 1) showed 1G2O1F indicating *BCR *deletion; group 4 (n = 2) with 1G1O1F indicating reciprocal *ABL1-BCR *deletion. Using the tri-colour dual fusion system, the added aqua-labelled *ASS1 *was found on the normal chromosome 9 and the der(9) but not in the der(22) (Figure 2, A – D, lower panel). In cases with der(9) deletion, either the *ASS1/ABL1 *signal or the *BCR *signal could be deleted. Similarly, in cases with reciprocal *ABL1-BCR *deletion, the aqua/orange/green signal was not detected on der(9). Nevertheless, the scoring of 'single-fusion' signal on der(22) in interphase cells remained specific and showed the same sensitivity as the use of D-FISH in classical 1G1O2F signal pattern. This was because genuine fusion would consist of orange and green signals without the aqua signal. In contrast, random signal overlap due to juxtaposition of chromosome 9 and 22 would show up as an orange/aqua/green signal. Thus, the potential false positivity of D-FISH in case of reciprocal *ABL1-BCR *deletion could be excluded. The tri-colour FISH was in fact, superior to the D-FISH system in the analysis of low numbers of interphase cells with reciprocal *ABL1-BCR *deletion that showed 1G1O1F signal pattern.

**Figure 3:**
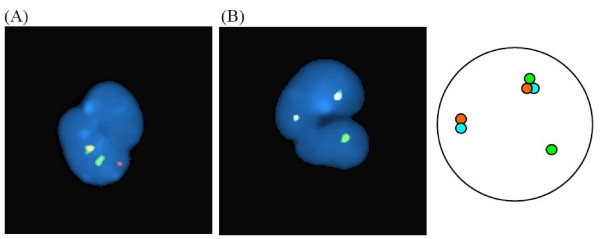
**Residual disease monitoring in patient with reciprocal *ABL1-BCR *deletion**. (A) Interphase BCR-ABL1 D-FISH shows random signal overlap that could be mistakenly scored as fusion signal. (B) Interphase BCR-ABL1 tri-colour dual fusion shows random signal overlap without genuine fusion.

The tri-colour dual fusion system was used to study a case with discrepant D-FISH and real-time quantitative reverse transcriptase polymerase chain reaction (RQ-RTPCR) results in a patient with reciprocal *ABL1-BCR *deletion. The patient was a 51 year-old male who was diagnosed with chronic myelogenous leukaemia in chronic phase in June 2006. He was commenced on 400 mg imatinib daily with complete haematological response and resolution of splenomegaly within 2 months. Interphase D-FISH on peripheral blood was used for residual disease monitoring. Major cytogenetic response was achieved within 6 months with 7% fusion signals by interphase D-FISH. After one year of imatinib treatment, the interphase D-FISH still showed 9% fusion signals. RQ-RTPCRwas performed, showing major molecular response with normalized *BCR-ABL1 *transcript level of 0.009% in the international scale. FISH was repeated using the tri-colour dual fusion system. Fusion signal was not found, thus indicating falsely positive D-FISH results tested previously (Figure 3, A – B).

## Discussion

Interphase FISH is being used increasingly for the diagnosis and therapeutic monitoring of CML. Despite the simplicity and rapid turn-around-time of the test, inappropriate interpretation of signal pattern may compromise the sensitivity of the test [6]. Technically, this can be due to poor hybridization signal or the presence of undesirable background. Moreover, the interpretation is sometimes difficult due to the inherent nature of signal integrity (e.g. split signals) and signal overlap. With the application of *BCR-ABL1 *D-FISH probe, the false positive rate due to signal overlap has been reduced to < 1% in comparison to the single fusion *BCR-ABL1 *probe [7].

Deletion of the *ABL1 *gene on der(9) has been shown to be a poor prognostic marker in CML [3,4] although its effect can be overcome by the use of imatinib [5]. Different types of submicroscopic deletion of the *BCR *and/or *ABL1 *have been well characterized with various *BCR-ABL1 *probe system [8,9]. However, most studies have focused on the fusion signal pattern on interphase cells and few have applied the *BCR-ABL1 *+ 9q34 tri-colour FISH probe system [1,10]. In this study, we have used D-FISH and tri-colour dual fusion system to study both metaphase and interphase signal patterns. The parallel analysis of both FISH system on metaphase has not been reported previously. Our result provides the basic groundwork towards the better understanding of the various *BCR-ABL1 *deletions and the correct interpretation of interphase FISH signals.

The D-FISH system is adequate for the diagnosis and therapeutic monitoring of CML with the classical t(9;22) (1G1O2F pattern) (Figure 2A, upper panel) and even for cases with *ABL1 *or *BCR *deletion, because in the latter, the single genuine fusion can be readily differentiated from random signal overlap due to the 'extra' green or orange signal (Figure 2B–C, upper panel). In addition, the D-FISH gives very reliable and specific delineation of the different signal patterns in metaphases (Figure 2A–D, upper panel). It is, however, difficult to differentiate random signal overlap from genuine fusion in cases with reciprocal *ABL1-BCR *deletion which will have 1G1O1F signal pattern in positive cells when tested by D-FISH (Figure 2D, upper panel). This single fusion pattern is often not a problem when the diagnostic sample is tested because the abnormal signal pattern is present in most interphase cells [11]. However, when interphase FISH is performed for disease monitoring, an atypical D-FISH result should be confirmed by the tri-colour dual fusion system. This is because the added aqua-labeled *ASS1 *probe consistently allows the discrimination of random signal overlap from genuine *BCR-ABL1 *fusion by the lack of aqua signal in the der(22) as the *ASS1 *is not involved in the t(9;22) and will remain in the der(9). The tri-colour dual fusion system is particularly useful in the study of reciprocal *ABL1-BCR *deletion where the signal pattern of 1G1O1F may sometimes be seen in a normal cell with random signal overlap. We have used the tri-colour dual fusion probe system to investigate the discrepant D-FISH and RQ-RTPCR result in a patient with reciprocal *ABL1-BCR *deletion. The tri-colour system did not show any fusion signal and the major molecular response was confirmed (Figure 3).

## Conclusion

While the D-FISH system can reliably detect *BCR-ABL1 *gene fusions, it is sometimes impossible to differentiate variant *BCR-ABL1 *and to exclude random signal overlap. For cases with atypical fusion signals due to events such as deletion of the *ABL1 *and/or *BCR*, complex *BCR-ABL1 *rearrangement and supernumerary Philadelphia (Ph), tri-colour dual fusion system can be used to further confirm the presence of *BCR-ABL1 *gene fusion. Finally, the tri-color dual fusion system should be recommended for monitoring residual disease in CML patients with der(9) deletion due to the superior analytical sensitivity over D-FISH in this group of patients.

## Competing interests

The authors declare that they have no competing interests.

## Authors' contributions

LLPS and ESKM conceived the study and were involved in the preparation of the manuscript. LLPS and KFW performed the test and analyzed the result. MHC and WSW provided the clinical information. All authors read and approved the final manuscript.

## Pre-publication history

The pre-publication history for this paper can be accessed here:



## References

[B1] Smoley SA, Brockman SR, Paternoster SF, Meyer RG, Dewald GW (2004). A novel tricolor, dual-fusion fluorescence in situ hybridization method to detect BCR/ABL fusion in cells with t(9;22)(q34;q11.2) associated with deletion of DNA on the derivative chromosome 9 in chronic myelocytic leukemia. Cancer Genet Cytogenet.

[B2] Herens C, Tassin F, Lemaire V, Beguin Y, Collard E, Lampertz S, Croisiau C, Lecomte M, De Prijk B, Longrée L, Koulischer L (2000). Deletion of the 5'-ABL region: a recurrent anomaly detected by fluorescence in situ hybridization in about 10% of Philadelphia-positive chronic myeloid leukaemia patients. Br J Haematol.

[B3] Sinclair PB, Nacheva EP, Leversha M, Telford N, Chang J, Reid A, Bench A, Champion K, Huntly B, Green AR (2000). Large deletions at the t(9;22) breakpoint are common and may identify a poor-prognosis subgroup of patients with chronic myeloid leukemia. Blood.

[B4] Huntly BJP, Reid AG, Bench AJ, Campbell L, Telford N, Shepherd P, Szer J, Prince H, Turner P, Grace C, Nacheva EP, Green AR (2001). Deletions of the derivative chromosome 9 occur at the time of the Philadelphia translocation and provide a powerful and independent prognostic indicator in chronic myeloid leukemia. Blood.

[B5] Huntly BJP, Guilhot F, Reid AG, Vassiliou G, Hennig E, Franke C, Byrne J, Brizard A, Niederwieser D, Freeman-Edward J, Cuthbert G, Bown N, Clark RE, Nacheva EP, Green AR, Deininger MWN (2003). Imatinib improves but may not fully reverse the poor prognosis of patients with CML with derivative chromosome 9 deletions. Blood.

[B6] Enns RK, Dewald G, Barker PE, Rasmussen DJ, Watson M, Wood G, Wyatt PR (2004). Fluorescence in situ hybridization (FISH) methods for medical genetics; approved guideline. NCCLS-MM7-A.

[B7] Dewald GW, Wyatt WA, Juneau AL, Carlson RO, Zinsmeister AR, Jalal SM, Spurbeck JL, Silver RT (1998). Highly sensitive fluorescence in situ hybridization method to detect double BCR/ABL fusion and monitor response to therapy in chronic myeloid leukemia. Blood.

[B8] Primo D, Tabernero MD, Rasillo A, Savagués JM, Espinosa AB, Chillón MC, Garcia-Sanz R, Gutierrez N, Giralt M, Hagemeijer A, San Miguel JF, Orfao A (2003). Patterns of BCR/ABL gene rearrangements by interphase fluorescence in situ hybridization (FISH) in BCR/ABL+ leukemias: incidence and underlying genetic abnormalities. Leukemia.

[B9] Kim YR, Cho HI, Yoon SS, Park S, Kim BK, Lee YK, Chun H, Kim HC, Lee DS (2005). Interpretation of submicroscopic deletions of the BCR or ABL gene should not depend on extra signal-FISH: problems in interpretation of submicroscopic deletion of the BCR or ABL gene with extra signal-FISH. Genes Chr Cancer.

[B10] Sinclair PB, Green AR, Grace C, Nacheva EP (1997). Improved sensitivity of BCR-ABL detection: a triple-probe three-color fluorescence in situ hybridization system. Blood.

[B11] Wolff DJ, Bagg A, Cooley LD, Dewald GW, Hirsch BA, Jacky PB, Rao KW, Rao PN (2007). Guidance for fluorescence *in situ *hybridization testing in hematologic disorders. J Mol Diagn.

